# Up-Regulation of leucocytes Genes Implicated in Telomere Dysfunction and Cellular Senescence Correlates with Depression and Anxiety Severity Scores

**DOI:** 10.1371/journal.pone.0049677

**Published:** 2012-11-21

**Authors:** Jean-Raymond Teyssier, Jean-Christophe Chauvet-Gelinier, Sylviane Ragot, Bernard Bonin

**Affiliations:** 1 Department of Genetics and Laboratory of Molecular Genetics, University Hospital, Dijon, France; 2 Psychiatry Unit, University Hospital, Dijon, France; 3 Laboratory of Psychopathology and Medical Psychology, University of Burgundy, Dijon, France; University of Newcastle, United Kingdom

## Abstract

**Background:**

Major depressive disorder (MDD) is frequently associated with chronic medical illness responsible of increased disability and mortality. Inflammation and oxidative stress are considered to be the major mediators of the allostatic load, and has been shown to correlate with telomere erosion in the leucocytes of MDD patients, leading to the model of accelerated aging. However, the significance of telomere length as an exclusive biomarker of aging has been questioned on both methodological and biological grounds. Furthermore, telomeres significantly shorten only in patients with long lasting MDD. Sensitive and dynamic functional biomarkers of aging would be clinically useful to evaluate the somatic impact of MDD.

**Methodology:**

To address this issue we have measured in the blood leucocytes of MDD patients (N = 17) and controls (N = 16) the expression of two genes identified as robust biomarkers of human aging and telomere dysfunction: p16^INK4a^ and STMN1. We have also quantified the transcripts of genes involved in the repair of oxidative DNA damage at telomeres (OGG1), telomere regulation and elongation (TERT), and in the response to biopsychological stress (FOS and DUSP1).

**Results:**

The OGG1, p16^INK4a^, and STMN1 gene were significantly up-regulated (25 to 100%) in the leucocytes of MDD patients. Expression of p16^INK4a^ and STMN1 was directly correlated with anxiety scores in the depression group, and that of p16^INK4a^, STMN and TERT with the depression and anxiety scores in the combined sample (MDD plus controls). Furthermore, we identified a unique correlative pattern of gene expression in the leucocytes of MDD subjects.

**Conclusions:**

Expression of p16^INK4^ and STMN1 is a promising biomarker for future epidemiological assessment of the somatic impact of depressive and anxious symptoms, at both clinical and subclinical level in both depressive patients and general population.

## Introduction

The excess of chronic (essentially cardiovascular and metabolic) medical illness in major depressive disorder (MDD), and the higher rate of associated disability and mortality [1]–[4], have led to the view that depression should be considered as both a psychic and a somatic disease [5]. There is evidence that pro-inflammatory mechanisms and systemic oxidative stress are key mediators of the “allostatic load”, and contribute to the two interlinked pathological dimensions of depression [6]–[9]. The impact of these factors on the cellular and organismal fate has been recently assessed by the measurement of the length of telomeres in blood leucocytes, which is a risk marker for age-related diseases, a longevity predictor at population level, and a biomarker of chronic oxidative stress [10]–[12]. In a preliminary study including 18 patients with MDD, Wolkowitz et al. [13] have shown that the mean telomere length (MTL), which was inversely correlated with the lifetime duration of the disease and with the degree of oxidative stress and inflammation, was significantly shorter in patients exposed for at least 9 years to depression. These authors have theorized that in vulnerable depressive subjects, telomere erosion translates the long lasting exposure of patients to allostatic mediators (including cytokines, steroids, and free radicals), which are responsible for accelerated cellular aging [14]. However, recent reviews and editorials have questioned the widespread use of leucocyte MTL as an exclusive biomarker of aging [15]–[17]. The validity of this indicator should be extensively assessed because of problems with standardization of the methodology, large inter-individuals variations, low statistical power of many cross-sectional studies, and the influence of unexplored confounding factors. In addition, the biological and epidemiological evidence that MTL is a surrogate measure of the normal aging process in adult individuals is still equivocal. More fundamentally, the intrinsic cell senescence mechanisms are not resumed by the progressive erosion of the single-stranded telomere overhang which activates the p53 – p21^cip1^ dependent senescence program [18]. In mammalian cells, the dysfunction of the sophisticated machinery formed by the capping proteins (the shelterins) and their interacting factors is a crucial determinant for senescence signaling from telomeres, which can be dissociated from telomere length [19]–[21]. Until recently, the stage of low level telomere dysfunction, which precedes MTL shortening in presenescent cells, was not detectable. Jiang et al. [22] have identified a set of four proteins, including stathmin encoded by the gene STMN1, which are secreted by aged cells with dysfunctional, but not critically short telomeres, from telomerase knockout mice, and whose levels linearly increased with age in the human blood plasma. On the other hand, experimental studies and correlative observations in humans, primates and rodents, have provided compelling molecular evidence that p16^ink4a^ encoded by the CDKN2A locus, plays a central role in the establishment and maintenance of the senescence state and is an effector of in vivo aging [18], [23]. The levels of p16^ink4a^ exponentially increase with age in most mammalian tissues. Its expression is up-regulated by diverse cellular stress including DNA damage and reactive oxygen species (ROS), and it is the downstream target of the so-called stress-activated Mitogen-Activated Kinases (p38MAPkinases). In human cells, the p16^ink4a^ dependent senescence mechanism is not specifically and acutely responsive to critical shortening of telomeres, and is activated independently of p53 [24]. However, p16^ink4a^ could also respond to the DNA damage repair signal by secondarily enforcing irreversible senescence, although the early activating events of the p16^ink4a^- pRb pathway during physiological aging remain to be elucidated [25], [26]. Consequently, the “telomere argument” in support of the “accelerated aging” theory in MDD needs to be further investigated and better grounded. For that purpose we have quantified the expression of the STMN1 and p16^ink4a^ genes, which may represent a sensitive and dynamic indicator in the blood leucocytes. In attempt to further explore the factors linking the psychological and cellular stress associated with MDD with the telomere system, we have also studied five other relevant genes (FOS, DUSP1, OGG1, IL-6, and TERT) whose expression can be considered putative biomarkers in the context of mood disorders. Integrated animal-human functional genomics studies have rated up-regulation of FOS and DUSP1 as a top candidate in anxiety and response to psychogenic stress [27]. The 8-oxoguanine-DNA glycosylase 1(OGG1) is the major repair enzyme of the Base Excision Repair (BER) pathway, which is a validated biomarker of oxidative stress and is required for the repair of oxidative guanine damage in telomeres and for maintaining telomere integrity in mammals [28], [29]. An increase in the plasma level of the pro-inflammatory cytokine IL-6 is associated with both MDD and aging [30], [31]. Finally, we assessed the expression of the gene encoding the catalytic subunit of the telomerase enzyme TERT. Changes in the activity of this key telomere regulating enzyme have been observed in MDD [32]. As a rule, expression of the TERT gene parallels telomerase activity [33]. The MTL and the transcripts of the genes have been measured in the peripheral blood leucocytes of female patients with MDD and of matched controls.

## Materials and Methods

### Participants

Seventeen female patients were recruited upon admission in the Psychiatry Unit (University Hospital of Dijon) for a depressive episode diagnosed by a psychiatrist with the Structured Clinical Interview for DSM-IV-TR (SCID) and the Mini-International Neuropsychiatric Interview (MINI). The severity of the disease has been assessed by the Hamilton severity depression and anxiety rating scales (HAM-D and HAM-A respectively). The life-long duration of the disease and the number of episodes, have been evaluated from clinical interviews and medical records. The diagnostic of MMD was established for the first time in 12 patients (first depressive episode). Five have experienced more than three episodes during a chronic duration of the disease ranging from 11 to 32 years (mean: 22.3±5.3). The duration of the present depressive episode was less than 6 months for all the patients. Subjects with any comorbid psychiatric disorder diagnosed by the MINI were excluded, excepted for anxiety symptoms. Medical examination, interview, medical records and laboratory tests were used to exclude somatic pathology, especially cardiovascular and metabolic. Five patients have history of tobacco use (at least five years) and were current smokers (5 to 20 cigarettes per day), and seven declared to drink moderately (mean: 2±0.6 alcohol units per week, range 0 to 6). None reported current use of drug of abuse. During clinical interview patients did not report severe adverse event during childhood, but this issue has not been systematically explored. Twelve patients received treatment by antidepressant and anxiolytic drugs (serotonin reuptake inhibitors and benzodiazepines), three patients were studied before treatment initiation, and the therapeutic status was unknown for two patients. The characteristics of these patients are provided in Table 1.

**Table 1:** Characteristics of the Depressed and Control Subjects.

	Controls (N = 16)	Depressed (N = 17)
**Age (Range)**	37.6±5.2 (23–45)	39.5±5.2 (22–54)
**Body Mass Index, kg/m^2^ (Range)**	23.8±5.6 (20–44.1)	23.3±5 (18.7–33.4)
**Alcohol consumption (units/week)**	30% (1.46±0.47)	31% (2±0.6)
**Current Tobacco use (cigarettes/day)**	0%	24% (14.6±1.3)
**Physical Activity, min/week (Range)**	108.1±42.3 (20–40)	71.5±53.9 (0–300)
**Chronicity of depression, years (Range)**	-	11.4±7.6 (0–32)
**Depressive episodes (Range)**	-	2±1.7 (1–6)
**HAM-D score (Range)**	2.8±0.6 (0–8)	25.3±4.3 (17–29)
**HAM-A score (Range)**	3.8±0.8 (0–10)	24.4±5 (15–37)

Sixteen control women recruited among hospital staff members were matched for age, BMI, physical activity, and alcohol consumption (mean 1.46±0.47 alcohol units per week, range 0 to 5). Controls were healthy active women without somatic disease, psychiatric diagnostic, medical treatment, or significant medical history (Table 1). All the included participants were of Caucasian origin.

### Ethics Statement

Patients and controls provided a written informed consent to participate in this study, and the detailed protocol was approved by the “Comité de Protection des Personnes” of the University Hospital of Dijon, in the framework of a Hospital Protocol of Clinical Research (N° 2009-A01146-51).

### Procedures

The steady state amounts of mRNA and the MTL have been assessed by the relative, real-time quantitative polymerase chain reaction method (rQ-PCR), as previously described [34], [35].

### Isolation of DNA and RNA from human circulating leukocytes

A total of 12 ml venous peripheral blood were collected from fasting patients and healthy controls at 8 a.m, in two 6 ml EDTA tubes. The RNA and DNA isolation procedures were performed within 2 hours after blood collection. The buffy coat containing most leucocytes was collected by centrifugation after lysis of red blood cells. The DNA was extracted by the standard salting-out technique, and its quality was checked by agarose gel electrophoresis. Total RNA was extracted from leukocytes using Trizol reagent (Life Technologies, USA) according to the manufacturer's protocol. The RNA samples were quantified using a NanoDrop 8000 spectrophotometer (Thermo Scientific, USA). Purity and quality were assessed by absorbance at UV_260/280_. A UV_260/280_ ratio greater than 1.8 was needed for further analysis. The quality of RNA, computed as RIN (RNA Integrity Number), was assessed with an Agilent 2100 Bioanalyzer (Agilent Technologies, USA) using RNA 6000 Nano Chips (Agilent Technologies, USA).

### Real-Time Quantitative-PCR for Gene expression

Each sample of total RNA was reverse transcribed using the QuantiTect Reverse Transcription Kit (Qiagen S.A., France) according to the manufacturer's protocol. The cDNA solution was then diluted fivefold, divided into aliquots and stored at −20°C. Two μl of the cDNA aliquots have been amplified by the Q-PCR technique using the QuantiTect SYBR Green PCR Kit (Qiagen S.A., France) following the manufacturer's instructions. All reactions were performed in a 10 μl mixture containing 5 µl SYBER Green Master Mix, 0.8 µl (80 ng) of each primer and 1.4 µl nuclease-free water. The Q-PCR reaction was carried out on a Light Cycler 480 II (Roche Diagnostics, France). The cycling conditions were the following: 95°C for 15 minutes (activation of HotStart Taq DNA polymerase), followed by 45 cycles of PCR amplification process including denaturing at 94°C for 15 seconds, annealing at 60°C for 1 minute and extension at 72°C for 40 seconds. A final step (95°C for 30 seconds, 50°C for 1 minute and gradual heating to 95°C) was set up to generate melting curves which checked the specificity of the amplification products. Each target and reference gene sequence has been amplified in triplicate wells for 45 cycles on the same plate. Negative controls (reverse transcription minus controls, cDNA replaced by nuclease-free water controls) were used to monitor nonspecific amplification. Standard curves derived from twofold serial dilutions of a template cDNA solution were generated to assess the reproducibility of the measured Ct values and to determine the PCR efficiency between 0.97 and 0.98. For each gene the control and MDD samples, the negative controls, and the standard curve dilutions have been amplified in the same 384-well plate. For each subject all the amplifications have been carried out using the same PCR reaction master mix and aliquots of the same cDNA sample. The results were expressed as the mean of the three Ct^GENE^ and Ct^GAPDH^ values (corresponding to the number of cycles producing a fluorescence signal above the threshold). The GAPDH gene has been selected as the reference (normalizing) gene after testing the homogenous expression of three genes (GAPDH, B2M, HGPRT) using the geNorm software [36]. The primers sequences (Table 2) were selected using the Primer3 software (Whitehead Institute for Biomedical Research) under stringent primer picking conditions (no 3′ self-complementarity and less than four self-complementarities). All the PCR primer pairs used generated amplicons between 80 and 150 base pairs.

**Table 2:** Sequences and localization on the CDS reference sequence (NCBI) of the forward (F) and reverse (R) primers used in the Q-PCR technique.

Genes	Sequences (3′>5′)	Sequence Reference	Localization
**FOS**	F actaccactcacccgcaga	NM_005252	291–309
	R ggaatgaagttggcactgg		372–390
**DUSP1**	F ttcctcaaaggaggatacgaag	NM_004417	606–627
	R actgcccaggtacagaaagg		784–803
**IL-6**	F gagtagtgaggaacaagccagag	NM_000600	518–540
	R gcgcagaatgagatgagttg		704–685
**OGG1**	F gcatcgtactctagcctccac	NM_002542	379–399
	R aggactttgctccctccac		481–500
**TERT**	F ccacgcgaaaaccttcct	NM_003219	2692–2709
	R agttcaccacgcagccatac		2737–2757
**STMN1**	F gttccagaattccccctttc	NM_005563	81–100
	R tttctcgtgctctcgtttctc		207–227
**P16^INK4a^**	F gcccaacgcaccgaatag	NM_000077	142–159
	R acgggtcgggtgagagtg		259–276
**GAPDH**	F tgcaccaccaactgcttagc	NM_002046	628–647
	R ggcatggactgtggtcatgag		694–714

### Real-Time Quantitative-PCR for Mean Telomere length

The mean telomere length (MTL) has been quantified by rQ-PCR method, exactly as previously described [37]. The published primers for the amplification of the repeated telomere sequence (T) were: (TelF) 5′GGTTTTTGAGGGTGAGGGTGAGGGTGAGGGTGAGGGT3′and (TelR) 5′TCCCGACTATCCCTATCCCTATCCCTATCCCTATCCCTA3′. Those for the amplification of the reference 36B4 single copy gene sequence (S) were: (36B4F) 5′CAGCAAGTGGGAAGGTGTAATCC3′ and (36B4R) 5′CCCATTCTATCATCAACGGGTACAA3′. The telomere and reference sequences were amplified in triplicate in two separate plates. Aliquots of 26.25 ng DNA were added in the master mix (QuantiTect SYBR Green PCR Kit, Qiagen S.A., France) containing either T-primers (TELF: 270 nM and TELR: 900 nM) or S-primers (36B4F: 300 nM and 36B4: 500 nM). For the generation of standard curves a reference human DNA sample was diluted serially to produce five concentrations of DNA (ranging from 0.6 ng/µl to 5 ng/µl), which were then distributed to the standard curve wells on each plate. The Q-PCR reactions were carried out on a Light Cycler 480 II (Roche Diagnostics, France). For the T (telomere) PCR, the cycling conditions were as followed: 95°C for 15 min (activation of HotStartTaq DNA polymerase), followed by 45 cycles of PCR amplification process including denaturing at 94°C for 15 s, annealing at 54°C for 2 min and extension at 72°C for 30 s. For the S (single gene copy) PCR, the cycling conditions were as followed: 95°C for 15 min (activation of HotStarTaq DNA polymerase), followed by 45 cycles of PCR amplification process including denaturing at 94°C for 15 s, annealing at 58°C for 1 min and extension at 72°C for 30 s. The specificity of the amplicons was checked by the melting curves obtained at the end of each PCR. The results were expressed by the mean Ct^T^ and Ct^S^ values.

### Statistical analysis

The difference (Δ) between the mean Ct values produced by the target sequences (Ct^GENE(1)^ for gene expression or Ct^T(1)^ for MTL measurement) normalized by the reference sequences (Ct^GAPDH(2)^ or Ct^S(2)^ respectively) in the test and control samples, is calculated by the same general formula: 2^−^(^(ΔCT(1)–ΔCS(2)^)  = 2^−ΔΔCt^ computed by the Relative Expression Software Tool (REST) [38]. Additionally, after the input of all individual mean Ct values, REST runs a powerful mathematical model for the determination of the statistical significance, which provides more robust data analysis and accurate “p” levels. This model corrects for PCR efficiency, tests for significance by an original Pair Wise Fixed Reallocation Randomisation Test, and plots using standard error estimation using a Taylor algorithm. For these reasons we used REST V2.0.7 for the comparison of both the gene expression levels and the MTL.

For the calculation of the Pearson's correlation coefficient |r|, and for linear regression analysis we used the Ct^GENE^/Ct^GAPDH^ and Ct^T^/Ct^S^ ratios as variables. Since the number of PCR cycles required to reach the threshold of the fluorescent PCR signal is inversely proportional to the initial amount of cDNA or DNA, it must be stressed that, acording to this current way of expressing the rQ-PCR data, the values of these ratios vary inversely with the transcript levels or the telomere length.

To facilitate the reading and understanding of the correlation curves we have used the negative value of the Ct ratios in the regression analysis. Between-group comparison for demographic variables was by the Student's t test. Statistical significance was fixed at p<0.05.

## Results

### Demographics

The depressed and control groups did not significantly differed regarding age (p = 0.76), BMI (p = 0.49), physical activity (p = 0.20) and alcohol consumption (p = 0.39). However, whereas 25% of the depressed subjects had history of tobacco use and were current smokers, none of the controls reported this habitus. The duration of the disease (chronicity) was directly correlated with the number of episodes (r = 0.66, p = 0.0001), as were the depression scores with the anxiety scores (r = 0.60, p = 0.045). There was no significant correlation between HAM-D or HAM-A scores and either the duration of the disease (p>0.6), the number of episodes (p>0.5), or any of the demographic factors summarized in Table 1 (all p>0.20).

### Mean telomere length and gene expression

The mean Ct values in the two populations are summarized in Table 3. In the population of women who predominantly have recently diagnosed MDD, the leucocyte MTL was not significantly different from that of controls. The relative MTL in the depressive group computed by REST was 1.123 (p = 0.67).

**Table 3:** The mean and standard deviation (inter-individual variability) of the Ct values in the depressed and control group. The I-WV value is the mean of the inter-well Ct variation (triplicate runs).

Gene	Controls			Depressed		
	Mean Ct	SD	I-WV	Mean Ct	SD	I-WV
**GAPDH**	17.32	0.46	0.116	18.29	0.82	0.100
**OGG1**	21.53	0.50	0.231	22.17	0.59	0.195
**STMN1**	17.45	0.12	0.215	17.61	0.14	0.202
**P16^INK4a^**	33.77	1.77	0.170	33.87	1.34	0.193
**TERT**	32.60	1.12	0.458	33.42	0.91	0.374
**DUSP1**	17.88	1;23	0.142	18.42	0.99	0.115
**FOS**	17.61	1.17	0.117	18.97	0.98	0.195
**IL6**	28.20	1.20	0.213	29.08	0.96	0.358
**Telomere**	13.60	0.30	0.078	13.42	0.32	0.053
**36B4**	21.86	0.16	0.076	21.87	0.32	0.050

Three genes were significantly overexpressed in the leucocytes of the MDD patients. The levels of the transcripts encoded by OGG1 were increased by 25%: relative expression level (REL) calculated by REST  = 1.249 (p = 0.04). The level of P16^ink4a^ expression doubled: REL = 1.993 (p = 0.002). The expression of STMN1 was increased by 65%: REL  = 1.647 (p = 0.002). There was also an increasing trend in the TERT transcript level: REL  = 1.311 (p = 0.05). There was no significant change in the REL of FOS (0.937, p = 0.9), DUSP1 (1.392, p = 0.18), or IL-6 (1.110, p = 0.68). It has been reported that markers of telomere dysfunction can be influenced by lifestyle factors including, smoking, physical activity and BMI (39). To assess the weight of these factors we performed a stepwise bidirectional multivariable regression analysis with clinical variables (Table 1) and the mean Ct ratios (for telomeres and all studied genes) as constant terms, and age, BMI, smoking status (yes or not, and number of smoked cigarettes per week), alcohol consumption, and physical activity as predictors. In the first model, the only potential confounding variable identified was age which directly correlated with the Ct ratio of P16^ink4a^ (r = 0.63, p = 0.03). After controlling for age, expression of P16^ink4a^ in the final best model only directly correlated with anxiety score (r = 0.71, p = 0.001). Furthermore, anxiety score did not correlate with age (F = 0.30, R^2^ = 0.29, p = 0.69). In this depressed group expression of P16^ink4a^, STMN1, OGG1 and TERT, did not correlated with smoking (all p>0.5), BMI (all p>0.5), or physical activity (all p>0.6). Multivariate regression analysis did not detect demographic confounder in the control group (all p>0.2). An inverse relationship between MTL and age approached the significance level only in the MDD subgroup (r = −0.14, p = 0.051). Except for this observation, in the whole population as well as in the depressed and control groups, the MTL correlated neither with any of the demographic and clinical variable described in the Table 1 (all p>0.2), nor with gene expression (all p>0.3).

**Figure 1:**
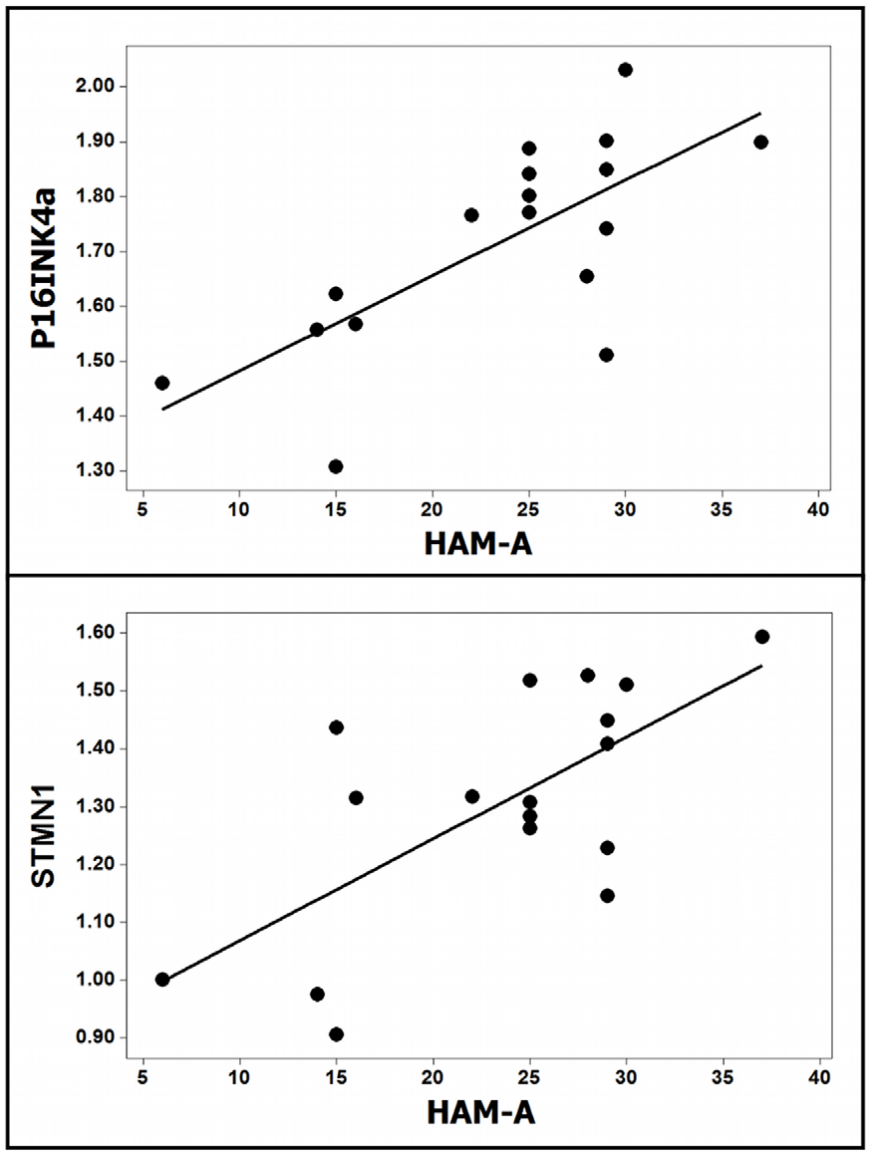
Adjustment curves of the linear regression analysis showing the direct correlation between the expression level of p16^INK4^ (F = 15.68, R^2^ = 0.51, p = 0.001) and STMN1 (F = 12.54, R^2^ = 0.45, p = 0.003) and the anxiety scores (HAM-A scale).

**Figure 2:**
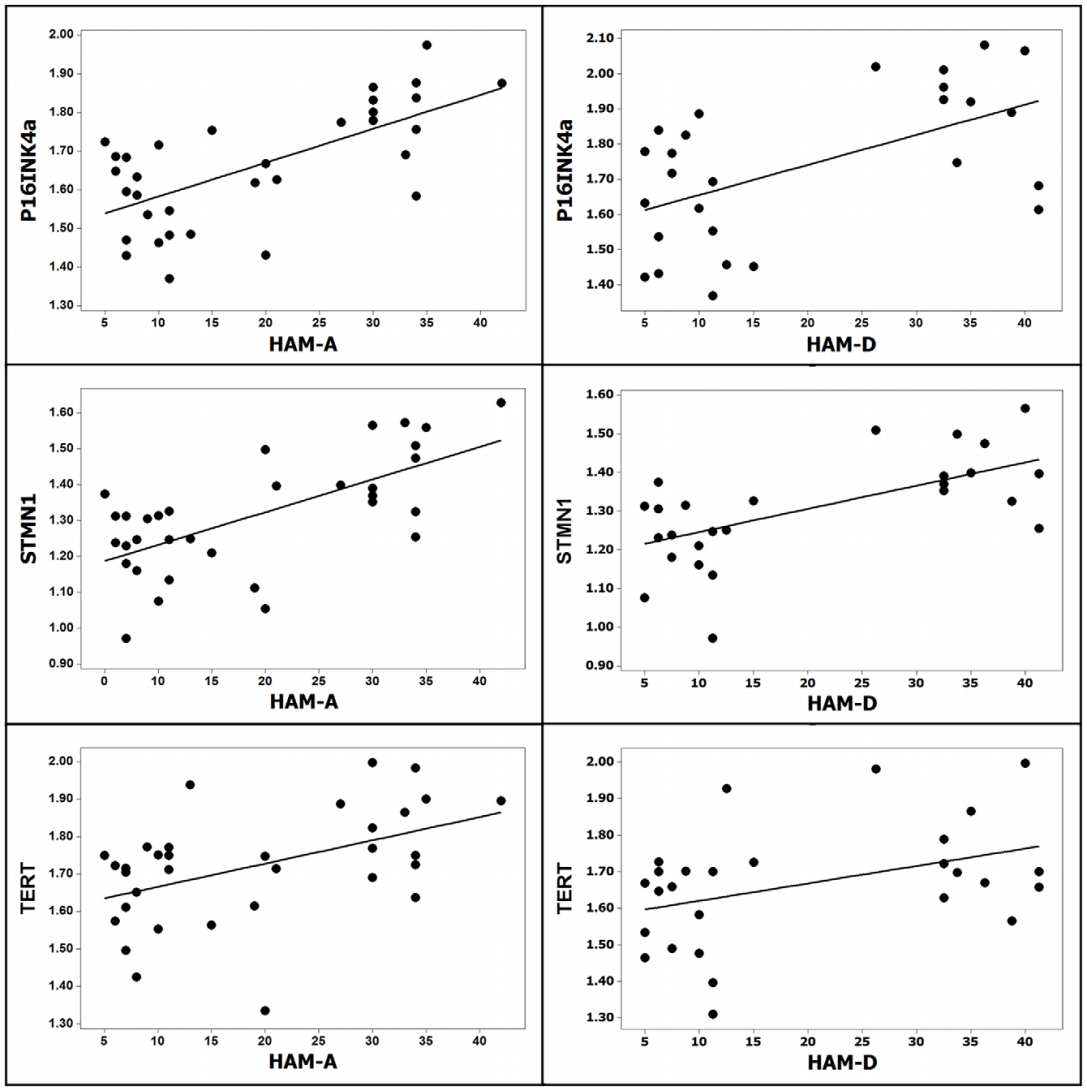
Adjustment curves of the linear regression analysis showing the direct correlation of HAM-A and HAM-D scores with gene expression in the combined sample (depressed plus controls): HAM-D with p16^INKa^ (F = 8.7, R^2^ = 0.27, p = 0.002), HAM-A with p16^INKa^ (F = 21.3, R^2^ = 0.43, p = 0.000), HAM-D with STMN1 (F = 15.7, R^2^ = 0.38, p = 0.001), HAM-A with STMN1 (F = 22.8, R^2^ = 0.44, p = 0.000), HAM-D with TERT (F = 4.9, R^2^ = 0.17, p = 0.03), HAM-A with TERT (F = 9.2, R^2^ = 0.23, p = 0.005).

In the MDD sample anxiety scores were strongly and directly correlated with the expression level of p16^INK4a^ (0.71, p = 0.001), STMN1 (0.67; p = 0.003), and non-significantly with that of TERT (0.47; p = 0.056). Unifactorial linear regression analysis disclosed that expression of STMN1 and p16^INK4a^ varied linearly and directly with the anxiety scores (Fig. 1), and that there was a trend for TERT (F = 4.30, R^2^ = 0.22, p = 0.05). There was no significant correlation between expression of the other genes and depression score, anxiety score, duration of the disease or number of episodes (all p>0.6). Regression analysis also highlighted a distinct coordinated pattern of gene expression in MDD subjects (Table 4). In the leucocytes from control subjects the levels of mRNA encoded by FOS and DUSP1 on the one hand, and of OGG1, IL-6, STMN1, and TERT on the other, were directly correlated. In contrast, in the leucocytes of MDD patients there was a generalized network of direct correlation embedding all the genes, including p16^INK4a^ and TERT. The analysis performed in the combined population of control and MDD subjects has extended these results to the spectrum of depression and anxiety scores, from normal to diagnostic levels. There was a moderate to strong direct linear relationship between the depression and anxiety scores and the expression of STMN1, p16^INK4a^ and TERT (Fig. 2).

**Table 4:** Correlative pattern of gene expression (Pearson's coefficient) in the blood leucocytes of controls and MDD subjects. For each gene the |r| value of control subjects (when significant) is in normal font weight on the upper row, and the |r| of the depressed patients in bold font weight on the lower row.

	DUSP1	IL-6	OGG1	STMN1	P16^INK4a^	TERT
**FOS**	0.54*					
	0.67**	0.64**	0.60***	0.41*	0.34*	0.59*
**DUSP1**		0.53*	0.81***	0.59**	0.52*	0.73*
**IL-6**			0.86***	0.66*		0.60*
			0.79***	0.56***	0.74**	0.80***
**OGG1**				0.70*		0.74*
				0.79**	0.78***	0.86***
**STMN1**						0.52*
					0.70***	0.79***
**P16^INK4a^**						0.76***

## Discussion

In this study we have reported for the first time that the expression of the two genes STMN1 and P16^ink4a^, which are markers of telomere dysfunction and cellular senescence, are up-regulated in the blood leucocytes of women with MDD. The STMN1 gene encodes stathmin, a ubiquitous cytosolic phosphoprotein proposed to function as an intracellular relay integrating regulatory signals of the cellular environment. This protein regulates formation of microtubules, and thus various cellular events depending on microtubules dynamics, including synaptic reorganization and plasticity [40]. In late generations of telomerase knockout mice (G4mTerc-/) with telomere dysfunction but not critically short MTL, aging cells secrete increasing amount of STMN1 which also accumulates in the blood plasma [22]. High amount of stathmin is also produced by presenescent human cells and after gamma irradiation, which supports the conception that telomere dysfunction, DNA damage and aging are intrinsically linked [22]. In humans, the strong correlation between the plasma level of the peptide and the chronological age allowed to discriminate young from old subjects in blind test samples [39]. This level also correlated with the expression of P16^ink4a^ and was influenced by lifestyle factors such as smoking and physical activity. The P16^ink4a^ coding sequence is part of the CDKN2A locus, which encodes several alternative-spliced variants involved in the cell cycle control. This peptide is a key tumor suppressor which inhibits the S-phase promoting activity of the cyclin-dependant kinases [41]. Moreover, genome wide association studies have consistently identified a single nucleotide polymorphism linked to this locus, which is a risk factor for age associated conditions like frailty and cardiovascular diseases [42], [43]. In rodent models and in primates including humans, P16^ink4a^ is strongly and specifically overexpressed during aging in most tissues and cells [44]–[46]. In an exploratory (N = 80) and a validation (N = 170) cohort of human donors aged 18–80 years, the level of P16^ink4a^ transcripts quantified by the Q-PCR technique lymphocytes increased exponentially with age (10 fold over six decades) [47]. By inducing irreversible cell cycle arrest in regenerative tissues and stem cells, up-regulation of P16^ink4a^ may play a causal role in tissue aging. It has been recently demonstrated in a transgenic mouse model, that clearance of P16^ink4a^–positive senescent cells could delay the age-related dysfunctional phenotypes [48]. Up-regulation of OGG1 in the leucocytes of MDD patients provides a substantial, although indirect, evidence for the role of oxidative stress in the pathologically activated aging process. The OGG1 gene encodes the bifunctional DNA glycosylase 1, which initiates the BER pathway [49]. The OGG1 selectively cleaves the principal oxidized base 8-hydroxy-2′-deoxyguanine (8-oxodG), which accumulates in the TTAGGG telomere repeats upon the action of ROS, causing DNA breakage and telomere erosion [50]. An increase in the level of serum of 8-oxodG has been previously reported in MDD and was correlated with severity and duration of depression [51]. Expression of OGG1 in the white blood cells is a recognized biomarker of in vivo exposure to oxidative stress, when physical analytical techniques are not sensitive enough to detect low level of oxidative DNA damage [28]. According to human and animal studies the BER pathway plays a crucial, although not fully elucidated role in aging and health span [52]. Deletion of OGG1 in yeast and mammalian cells results in base damage and telomere dysfunction [29], [50]. Finally, It is noteworthy that a large scale genetic linkage study including more than 800 multiplex families and applying a pathway and network ranking method, has identified the loci of OGG1 and STMN1 in a cluster of interacting genes associated with schizophrenia, those of TERT and CDKN2A in a cluster associated with autism, and that of CDKN2A in a cluster associated with bipolar disorder [53]. This pattern strengthens the view that the action of the genes involved in psychiatric diseases is intrinsically pleiotropic.

We failed to detect telomere shortening or any correlation of MTL with gene expression or clinical variables in our sample of women, with predominantly recently diagnosed MDD. Previous studies which have measured MTL in the leucocytes from patients with MDD have reported that telomere shortening only occurred in patients with a long lasting (10 to 30 years) depressive disease [13], [54]–[56]. Collectively, these data and the results of the present study give strength to the hypothesis that MDD is a pathological condition which triggers telomere dysfunction and activates the senescence pathway in blood leucocytes at an early phase of its evolution. The MTL represents a cumulative marker of a long-lasting exposure to pathological conditions, including stressful events, inflammation and oxidative stress [57]. It does not provide an adequate tool for monitoring the impact of MDD throughout its course. We would suggest that the quantification of the expression of STMN1 and P16^ink4a^ by the Q-PCR technique could provide a test which fulfills this purpose, since these genes meet some fundamental criteria defining biomarkers of molecular aging [31]. This test which has low intra-individual variability, is sensitive, highly dynamic with human age, can be easily standardized, and provides functional information. Its clinical and biological significance, especially as a risk factor for premature age-related medical diseases in psychiatric disorders, should be now systematically evaluated by longitudinal prospective studies. Furthermore, the correlation of the expression of P16^INK4A^ and STMN1 with anxiety scores in MDD, and with scores of depression and anxiety in the combined population, raises the possibility that quantification of these gene transcripts in blood leucocytes could be used to explore the dimensional components of the stress load associated with depressive and anxious symptoms in both clinical and community samples.

By using correlation analysis to statistically infer a gene co-expression pattern, we were able to identify a network in the leucocytes of MDD patients, not observed in control subjects. This encompassed all the studied genes, specifically including P16^ink4a^ and the FOS-DUSP1 module (which was unconnected to the other genes in cells from controls). It is remarkable that the strongest |r| values (>0.7) were assigned to the expression of OGG1 (correlation with the five other genes) and TERT (correlation with STMN1, DUSP1, and P16^ink4a^). This observation indirectly confirms the relationship between oxidative DNA damage, telomere dysfunction and cell senescence in the leucocytes of MDD patients. On the other hand, the correlation linking the expression of the FOS-DUSP1 module to that of the other genes, only observed in MDD leucocytes, suggests that MAPKs signaling played a role in the response of these cells to MDD condition. Both FOS and DUSP1 are immediate-early genes implicated in the stress-responsive p38MAPK pathway, which among other things activates P16^ink4a^. The FOS peptide is a constitutive part of the AP-1 transcription factor, which ubiquitously controls the cellular gene expression profiles. The DUSP1 gene encodes a dual specificity protein phosphatase which, by efficiently inactivating the MAPKs, contributes to the termination of the phosphorylation cascade in a feed-back control loop [58], [59]. To identify and prioritize candidate genes in anxiety and sensitivity to psychogenic stress, Le-Niculescu et al. [27] have used a convergent, integrated functional genomics approach. Changes in gene expression detected by microarrays in animal experimental behavioral and pharmacologic models of these two conditions, have been cross-validated by a comprehensive database of all the published human and animal genetic and expression results in psychiatric diseases (especially in mood disorder, post-traumatic stress disorder, and obsessive compulsive disorder). After evidence scoring the FOS has been ranked the top gene, and DUSP1 was included in the list of the six top candidates. Similarly, a previous study of the monocyte transcriptome from subjects with atherosclerosis had shown that up-regulation of FOS and DUSP1 was a reliable biomarker of disease severity (especially of the inflammatory state) [60]. Although TERT expression was not significantly increased in the leucocytes of depressive patients, it was strongly correlated with that of the others genes, and was associated with the anxiety and depressive dimensions. These findings are consistent with the recent study by Wolkowitz et al. [32], who reported that the catalytic activity of telomerase was elevated in mononuclear cells of patients with long lasting MDD, and that this activity which was directly correlated with depressive ratings influenced the antidepressant response. Thus telomerase activation may represent a potential new marker of the somatic impact of MDD. The pathophysiological significance of TERT remains ellusive, since this protein is involved in various separate functions uncoupled with telomere elongation, including telomere capping, DNA repair, the control of cell lifespan, and the regulation of transcription [61].

It must be stressed that the biological significance of these findings is still unresolved. Whether the described changes are limited to the inflammatory cell compartment, or also affect other tissues which are the targets of the aging process such as brain, kidney and arteries, remains to be determined. We have recently shown that telomere shortening and activation of the stress response genes including OGG1 do not occur in the cortex of patients with MDD [62]. This observation is worth considering since over-expression of OGG1 is a hallmark of the normal human aging brain [63]. Finally the association of up-regulation of STMN1 with the HAM-A scores raises a potential important issue. This gene is highly expressed in the amygdala and associated thalamic and cortical structures which control the fear and anxiety response. In *stathmin* knockout mice, a decrease in microtubules dynamics in the amygdala was associated with a blunted fear response to both learned and innate stimuli [63]. In humans, predominantly in women, there is experimental evidence that a single nucleotide polymorphism located in the upstream regulatory region of the STMN1 gene modulates the reactivity to stressful and fearful stimuli [64], but also is involved in cognitive and affective control processes [65], [66]. This observation suggests the possibility of an intriguing pleiotropic mechanism, according to which up-regulation of STMN1 in blood leucocytes might be both a marker of cell senescence and a peripheral surrogate of the dysfunctional neuronal networking.

The strength of the present study is the use of middle-aged female patients, with predominantly short lasting MDD without notable confounding factors. The major limitation is the small sample size which did not allow the analysis of sub-samples, and prevented the detection of inter-group differences of moderate effect size, whose reality is suggested by the extensive observed correlations. Additionally, as noticed in the previous related publications, it remains to be established that changes in the composition of the peripheral populations of blood leucocytes do not partially account for such results. Since most patients were treated by antidepressant and anxiolytic drugs, we cannot exclude the influence of these therapeutic regimens.

### Conclusion

In this preliminary study we have showed that MDD is associated with up-regulation of the p16^INK4a^ and STMN1 genes, which is a robust marker of telomere dysfunction and biological aging. This observation strengthens the “accelerated aging” model [14], and suggests that these markers may have clinical application to monitor the course and somatic consequences of MDD, but also to evaluate the effects of the subclinical anxious and depressive spectrum in general population.
